# A New X-ray Medical-Image-Enhancement Method Based on Multiscale Shannon–Cosine Wavelet

**DOI:** 10.3390/e24121754

**Published:** 2022-11-30

**Authors:** Meng Liu, Shuli Mei, Pengfei Liu, Yusif Gasimov, Carlo Cattani

**Affiliations:** 1 College of Information and Electrical Engineering, China Agricultural University, Beijing 100083, China; 2 Huiying Medical Technology Co., Ltd., Beijing 100192, China; 3 Department of Mathematics and Informatics, Azerbaijan University, AZ1007 Baku, Azerbaijan; 4 Institute of Mathematics and Mechanics, ANAS, B. Vahabzade Str., 9, AZ1148 Baku, Azerbaijan; 5 Institute of Physical Problems, Baku State University, Z. Khalilov, 23, AZ1148 Baku, Azerbaijan; 6 Engineering School, DEIM, University of Tuscia, 01100 Viterbo, Italy

**Keywords:** Shannon–Cosine wavelet multiscale decomposition, DR medical images, image enhancement, inverse sharpening mask

## Abstract

Because of noise interference, improper exposure, and the over thickness of human tissues, the detailed information of DR (digital radiography) images can be masked, including unclear edges and reduced contrast. An image-enhancement algorithm based on wavelet multiscale decomposition is proposed to address the shortcomings of existing single-scale image-enhancement algorithms. The proposed algorithm is based on Shannon–Cosine wavelets by taking advantage of the interpolation, smoothness, tight support, and normalization properties. Next a multiscale interpolation wavelet operator is constructed to divide the image into several sub-images from high frequency to low frequency, and to perform different multi-scale wavelet transforms on the detailed image of each channel. So that the most subtle and diagnostically useful information in the image can be effectively enhanced. Moreover, the image will not be over-enhanced and combined with the high contrast sensitivity of the human eye’s visual system in smooth regions, different attenuation coefficients are used for different regions to achieve the purpose of suppressing noise while enhancing details. The results obtained by some simulations show that this method can effectively eliminate the noise in the DR image, and the enhanced DR image detail information is clearer than before while having high effectiveness and robustness.

## 1. Introduction

The enhancement of medical images is a task of high practical value. In fact, many current medical images, especially X-ray DR images of low-dose projection data, are often blurred in the original image [1,2]. In medicine, these blurred images contain a lot of important details and information that is crucial for medical diagnoses. Therefore, detail enhancement for medical images has been one of the main focuses of research [3,4].

In the area of improving image quality, there are three main issues to be addressed, namely contrast enhancement, noise reduction, and image sharpening. The most common image-enhancement methods are based on histogram equalization (HE) [5], contrast-limited adaptive histogram equalization (CLAHE) [6,7,8], and morphological algorithms [9,10]. Histogram equalization algorithms have been shown to be a viable option for medical-image enhancement. By using a cumulative distribution function (CDF), gray levels are mapped from low-contrast images to obtain an enhanced gray-scale image. Although the HE method is simple to calculate, high peaks in the histogram can over enhance the image, introducing artifacts and noise so that structural details may be eliminated or reduced. Ismail et al. [6] and Isa et al. [7] introduced the adaptive blurred histogram equalization scheme for magnetic resonance (MR) image enhancement. This scheme is achieved by normalizing and smoothing the histogram of the input image, followed by sub-image HE processing. This method preserves the average brightness in the MR images of the brain. Although CLAHE has been widely used, its performance in and the quality of the enhanced images are highly dependent on the choice of block size, the number of histogram slots, the intensity range of the enhanced images, the specified distribution of image blocks, and the parameters of the distribution itself. Unlike traditional feature-learning methods, using optical flow field and patch-based normalized cross-correlation (NCC) data terms, the optical flow field can ensure that global optimization can more effectively compress the noise within MR images than can other optical flow methods through a special regularization term, overcoming the lack of relatively homogeneous organization in bright gradients, which can effectively enhance images, but the high time cost cannot be ignored [11]. Another algorithm for enhancing medical images is the proposed morphological operation that gives satisfactory results. Unsharp masking (UM), which is based on morphological operations [12], is a common algorithm for image detail and edge enhancement. In the inverse sharpening masking algorithm, the difference in the activity of the pixels is exploited to double-enhance the image using the Laplace operator for second-order differences [13]. Linear image enhancement is simple in principle and fast in execution, but the results are not ideal, and some image detail is usually lost because of uneven image enhancement. In summary, a good medical-image-enhancement algorithm should have, at the same time, properties such as the ability to suppress noise during image enhancement, the ability to enhance the contrast of the image without losing brightness, and the ability to quickly and reliably be set up.

Textures in medical images present mostly irregular, smooth, and closed-curve structures. On the other hand, existing image-enhancement methods focus only on solving individual problems on an image, such as increasing contrast, reducing noise, and/or increasing sharpness. Therefore, ensuring that improvements on image problems are parallel is of great significance for many image-processing applications. In contrast, the wavelet transform has the advantages of easy noise removal, ease of operation, and the ability to reflect information on image feature points [14,15,16]. In this work, in order to achieve a dynamic capture and an accurate representation of dynamic curve features, we first use the fluctuation and continuity of the Shannon wavelet function to design a parametric window function according to the integral median theorem, and then through parameter adjustment, we can meet the requirements for the adaptive control of the Shannon–Cosine wavelet on the support interval and smoothness, so as to achieve the texture of medical images The result is a parametric window function that can be adapted to meet the requirements of the Shannon–Cosine wavelet on the support interval and smoothness, to achieve texture approximation in medical images. Some of the main contributions of this paper are the following:

1. In the framework of the algorithm of Shannon–Cosine wavelet multiscale decomposition, a digital X-ray image-enhancement curve was designed according to the characteristics of noise in the image, which can effectively suppress quantum noise while enhancing the image and can ensure that the overall enhancement effect will not be overshot while enhancing the details.

2. We designed a digital X-ray noise-reduction algorithm based on the pixel activity in different regions, which is based on the pixel activity. This algorithm can maintain the diagnostic details better than the conventional algorithm can, while smoothing the unstructured regions.

The organization of this paper is as follows: in Section 2 some preliminary remarks about multiscale analysis by Shannon–Cosine are provided; Section 3 describes the algorithm for a multiscale digital X-ray image-enhancement and noise-reduction algorithm; Section 4 features adaptive multiscale enhancement for and a noise-reduction simulation for DR images; and in Section 5, a discussion on future perspectives and conclusions is provided.

## 2. Wavelet Multiscale Transform Fundamentals

The method of solving nonlinear partial differential equations is a common method for medical-image processing, which can achieve edge-preserving noise reduction in images, but the method lacks multiscale characteristics and therefore has poor protection for fine textures. After the difference method or the single-scale wavelet numerical method to solve two-dimensional partial differential equations is used, the total number of discrete points is large, which hinders the solution of large data volume solving problems (such as image processing) in engineering. Therefore, it is of great importance to extend the application of the wavelet fine integration method to solving two-dimensional partial differential equations. The key to constructing the wavelet fine integration algorithm for two-dimensional partial differential equations is constructing a two-dimensional multiscale interpolation wavelet operator. By adaptively discretizing the partial differential equations by using the multiscale wavelet interpolation operator, the obtained set of ordinary differential equations can be solved: by directly using the adaptive fine integration method, based on the extrapolation technique.

### 2.1. Shannon–Cosine Wavelet and Their Properties

The main features of Shannon–Cosine wavelet function are interpolation, smoothness, compact support, and symmetry [17,18]. Shannon–Cosine wavelet mother wavelet function are defined as:

(1) $$\varphi (x)=\frac{\sin(\pi x)}{\pi x}\sum_{n=0}^{m}\left(a_{n}\cos\left(\frac{2n\pi x}{N}\right)\right)\cdot \;\left(\chi \left(x+\frac{N}{2}\right)-\chi \left(x-\frac{N}{2}\right)\right)$$

where N is a constant associated with the support interval and χ(x) is the Heaviside function. This function is defined as:

(2) $$\chi (x)=\left\{ \begin{array}{ll} 0 & x<0\\ undefined & x=0\\ 1x>0 & x>0 \end{array}\right.$$

where the support interval of the function is [−N/2, N/2]. Coefficients $a_{i}(i=0,1,\ldots ,m)$ are used to fulfill the smoothness at the boundary, which can be obtained by applying the following set of differential equations:

(3) $$\frac{d^{n}}{dx^{n}}\phi \left(\frac{N}{2}\right)=0,\;\;n=0,1,\ldots ,m$$

**Figure 1:**
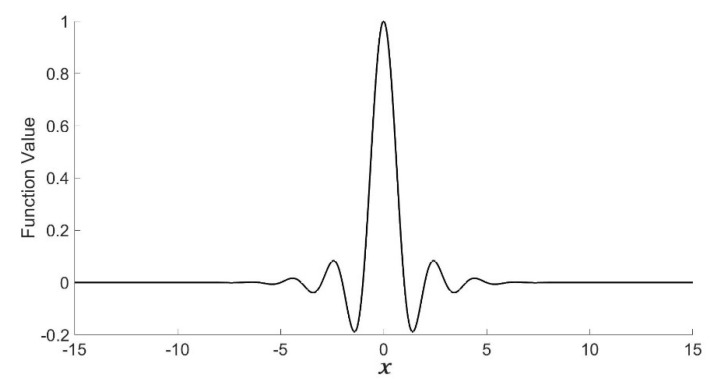
Parameterized Shannon–Cosine function.

Here it is not difficult to verify whether the mother wavelet function of the multiscale Shannon–Cosine wavelet has the interpolation property that ϕ(0)=1 [19]. By taking *x* = *N*/2 (or *x* = −*N*/2) and *x* = 0 into Equations (1) and (3), we obtain a system of linear algebraic equations with respect to the coefficients. Figure 1 illustrates the Shannon–Cosine wavelet-generating function image.

Support interval parameter N can be found from normalization condition $\int_{-\infty }^{\infty }\phi (x)dx=1$ of the wavelet’s parent function, and the choice of parameter N is related to the waveform of the Shannon–Cosine wavelet, which can find functions with an integral greater than 1 in one interval and less than 1 in another interval. This shows that making a reasonable choice on a support interval can ensure that the parameterized Shannon–Cosine polynomial function satisfies the uniformity condition. Unlike the Shannon–Gabor wavelet, the Shannon–Cosine wavelet is a true tight support wavelet that meets all the definitions of a wavelet and contributes to the efficiency and numerical accuracy of the algorithm.

### 2.2. Multiscale Interpolation Wavelet Operator

Let ϕ(x) be the wavelet mother function with the interpolation property, and the sequence of functions obtained by translation and scaling is defined as:

(4) $$\phi_{j,k}=\phi (2^{j}x-k)$$

where $\phi_{j,k}$ is the scale basis function, $k=0,1,2,\ldots ,2^{j};j\in z$, j is the scaling factor, and k is the translation factor. For functions $f(x)\in L^{2}(0,1)$ and $x\in \left[x_{\min},x_{\max}\right]$, the interpolated wavelet transform coefficients are defined as:

(5) $$\alpha_{j,k}=f(x_{j,k})-\left(\sum_{k_{0}=0}^{2^{j_{0}}}f(x_{j_{0,}k_{0}})\phi_{j_{0},k_{0}}(x_{j,k})+\sum_{j_{1}=j_{0}}^{j-1}\sum_{k_{1}=0}^{2^{h}-1}\alpha_{j_{1},k_{1}}\psi_{j_{1},k_{1}}(x_{j,k})\right)$$

where $\psi_{j,k}(x)=\phi_{j+1,2k+1}(x)$ is the wavelet definition function, $\Delta x_{j}=\frac{x_{\max}-x_{\min}}{2^{j}}$, $\Delta x_{J}=\frac{x_{\max}-x_{\min}}{2^{J}}$, $x_{j,k}=x_{\min}+k\Delta x_{j}$, and $x_{J,n}=x_{\min}+n\Delta x_{J}$.

Based on the above definition, the definition of the multiscale interpolation wavelet transform matrix, $C_{k,n}^{j,J}$, can be given, where $k\in \left\{0,1,2,\ldots ,2^{j}\right\}$, $0\leq j_{0}\leq J-1$, and $n\in \left\{0,1,2,\ldots ,2^{J}\right\}$. According to the definition of multiscale wavelet transform, the wavelet transform is therefore obtained [20]. The corresponding wavelet coefficients are:

(6) $$\alpha_{j,k}=\sum_{n=0}^{2^{J}}C_{k,n}^{j,J}f(x_{J,n})$$

According to the definition of interpolated wavelet transform coefficients, we have:

(7) $$\alpha_{j,k}=f(x_{j,k})-\left(\sum_{k_{0}=0}^{2^{j_{0}}}f(x_{j_{0},k_{0}})+\sum_{j_{1}=j_{0}}^{j-1}\sum_{k_{1}=0}^{2^{j_{1}}-1}\alpha_{j_{1},k_{1}}\psi_{j_{1},k_{1}}(x_{j,k})\right)$$

where $\psi_{j,k}=\phi_{j+1,2k+1}$. When the definition of the restriction operator is used, it is not difficult to obtain:

(8) $$\left\{ \begin{array}{l} f(x_{j,k})=\sum_{n=0}^{2^{j}}R_{2k+1,n}^{j+1,J}f(x_{J,n})\\ f(x_{j_{0},k_{0}})=\sum_{n=0}^{2^{j}}R_{k_{0},n}^{j_{0},J}f(x_{J,n}) \end{array}\right.$$

By substituting Equation (8) into Equation (7), we get:

(9) $$\alpha_{j,k}=\sum_{n=0}^{2^{J}}\left(R_{2k+1,n}^{j+1,J}-\sum_{k_{0}=0}^{2^{j_{0}}}R_{k_{0},n}^{j_{0},J}\phi (x_{j+1,2k+1})\right)f(x_{J,n})-\sum_{j_{1}=j_{0}}^{j-1}\sum_{k_{1}=0}^{2^{j_{1}-1}}\alpha_{j_{1},k_{1}}\psi_{j_{1},k_{1}}(x_{j+1,2k+1})$$

By substituting Equation (6) into Equation (9), we get:

(10) $$\begin{array}{ll} \sum_{n=0}^{2^{J}}C_{k,n}^{j,J}f(x_{J,n}) & =\sum_{n=0}^{2^{J}}\left(R_{2k+1,n}^{j+1,J}-\sum_{k_{0}=0}^{2^{j_{0}}}R_{k_{0},n}^{j_{0},J}\phi_{j_{0},k_{0}}(x_{j+1,2k+1})\right)f(x_{J,n})\\ & \;-\sum_{n=0}^{2^{J}}\sum_{j_{1}=j_{0}}^{j-1}\sum_{k_{1}=0}^{2^{j_{1}-1}}C_{k_{1},n}^{j_{1},J}f(x_{J,n})\psi_{j_{1},k_{1}}(x_{j+1,2k+1})\\ & =\sum_{n=0}^{2^{J}}\left(R_{2k+1,n}^{j+1,J}-\sum_{k_{0}=0}^{2^{j_{0}}}R_{k_{0},n}^{j_{0},J}\phi_{j_{0},k_{0}}(x_{j+1,2k+1})\right)f(x_{J,n})\\ & \;-\sum_{n=0}^{2^{J}}\left(\sum_{j_{1}=j_{0}}^{j-1}\sum_{k_{1}=0}^{2^{j_{1}-1}}C_{k_{1},n}^{j_{1},J}\psi_{j_{1},k_{1}}(x_{j+1,2k+1})\right)f(x_{J,n})\\ & =\sum_{n=0}^{2^{J}}\left(R_{2k+1,n}^{j+1,J}-\sum_{k_{0}=0}^{2^{j_{0}}}R_{k_{0},n}^{j_{0},J}\phi_{j_{0},k_{0}}(x_{j+1,2k+1})-\sum_{j_{1}=j_{0}}^{j-1}\sum_{k_{1}=0}^{2^{j_{1}-1}}C_{k_{1},n}^{j_{1},J}\psi_{j_{1},k_{1}}(x_{j+1,2k+1})\right)f(x_{J,n}) \end{array}$$

It is not difficult to obtain the Shannon–Cosine wavelet-based multiscale interpolated wavelet transform matrix by comparing the expressions on both sides of the equal sign in Equation (10):

(11) $$C_{k,n}^{j,J}=R_{2k+1,n}^{j+1,J}-\sum_{k_{0}=0}^{2^{j_{0}}}R_{k_{0},n}^{j_{0},n}(x_{j+1,2k+1})-\sum_{j_{1}=j_{0}}^{j-1}\sum_{k_{1}=0}^{2^{j_{1}-1}}C_{k_{1},n}^{j_{1},J}\psi_{j_{1},k_{1}}(x_{j+1,2k+1})\;C_{k,n}^{j_{0},J}=R_{2k+1,n}^{j_{0}+1,J}-\sum_{k_{0}=0}^{2^{j_{0}}}R_{k_{0},n}^{j_{0},J}\phi_{j_{0},k_{0}}(x_{j_{0}+1,2k+1})$$

where $R_{k,n}^{j,J}=\left\{ \begin{array}{l} 1,x_{j,k}=x_{J,n}\\ 0,otherwise \end{array}\right.$ is the restriction operator.

**Figure 2:**
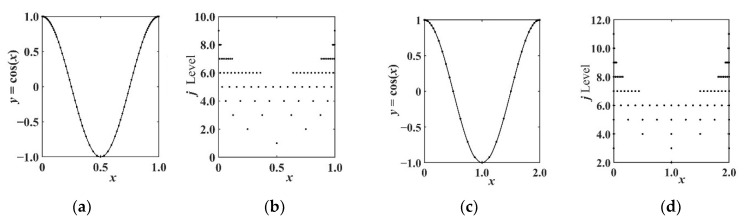
Constructing Shannon–Cosine wavelet multiscale interpolation curves. (**a**) Shannon-Cosine wavelet approximation. (**b**) Stratified distribution of 60 feature points. (**c**) Interval Shannon-Cosine wavelet approximation. (**d**) Stratified distribution of 40 feature points.

Based on the above analysis, the multiscale adaptive subdivision interpolation results of the curve made by the Shannon–Cosine wavelet are shown in Figure 2.

As shown in Figure 2a,b, when the cosine curve is adaptively subdivided by using the Shannon–Cosine wavelet method, more feature points are concentrated at the endpoint of the curve. When the curve is subdivided by using the Shannon–Cosine interval wavelet method, as shown in Figure 2c,d, more subdivision features are also concentrated at the boundary points of the curve. In this work, the Shannon–Cosine wavelet function is used to discretize the partial differential equations, by using the multiscale nature of wavelets to capture image-texture features, allowing for sparse points in smooth areas and dense points in rich texture details, thus effectively reducing the number of equation sets while maintaining the texture of the image. Finally, the wavelet fine integration method is used to solve the ordinary differential equations. The high-precision solution is obtained, and the solution of the equation set is the pixel value of the image at that point after noise reduction.

## 3. Multiscale Digital X-ray Image-Enhancement and Noise-Reduction Algorithm

In this paper, we take advantage of the multiscale properties of the Shannon–Cosine wavelet to propose an enhancement algorithm for medical X-ray images. The algorithm system consists of four major parts: (1) imaging for DR images, (2) Shannon–Cosine wavelet decomposition and reconstruction for DR images, (3) multiscale diagnostic detail enhancement for DR images, and (4) simulations for multiscale noise reduction in DR images.

**Figure 3:**
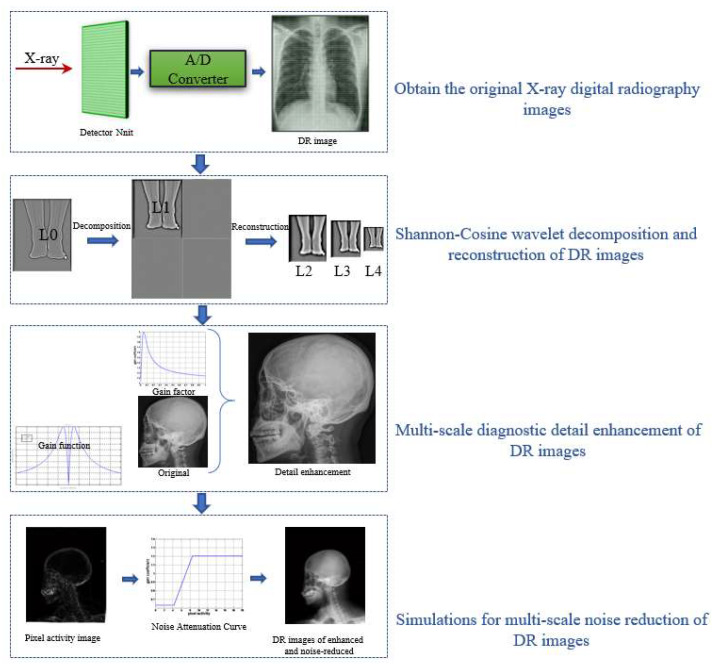
Image-enhancement system.

Figure 3 shows the basic algorithm framework, corresponding to the following steps:

^1.^  X-rays are passed through the analog-to-digital (A/D) converter to obtain the original X-ray digital photography image, $f(x_{j,k})$.
^2.^  The Shannon–Cosine wavelet transform is applied to the original image by using the multiscale properties of the Shannon–Cosine wavelet. A set of high-frequency images, L0, L1, L2,… Ln, and a low-frequency image, gn, are obtained after multilayer decomposition.
^3.^  A nonlinear gain function,
  $$y=\frac{x}{A+B\cdot x+C\cdot x^{p}},\;x>0$$
  is designed to control the degree of enhancement for the large dynamic range of digital X-ray images. The gain coefficient is multiplied by the gray value of each pixel at different scales to change the detail image, and then the image is reconstructed to achieve the enhancement effect.
^4.^  The degree of pixel activity $v_{i}(n,m)$ is defined by calculating the standard deviation within the local neighborhood of the central pixel. The pixel activity level is then bilinearly interpolated to correspond to high-frequency images at different scales, Li. Finally, the pixel gray-scale values are attenuated and enhanced according to the strength of each pixel’s activity level to achieve noise reduction.

### 3.1. Multiscale Diagnostic Detail Enhancement on DR Images

**Figure 4:**
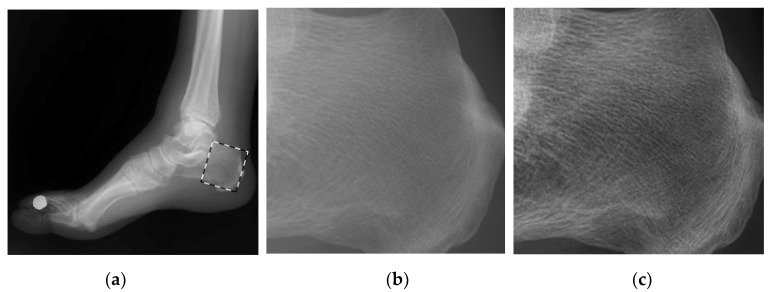
Schematic of detail enhancement. (**a**) Foot image after hue transformation (**b**) foot image after partly enlarged display (**c**) foot image after detail enhancement.

In DR image processing, there are often some finer details that enable physicians to be more precise about diagnosis, but due to the large dynamic range of the whole image, this information may not be seen very clearly when displayed. Therefore, it needs to be enhanced before display. It is important to the enhancement of fine details in the detail space of the image while preventing the noise from being overly amplified. As shown in Figure 4.

The specific method of image enhancement is to first decompose it into a set of high-frequency images and a low-frequency image, and then multiply different pixel gray values in the high-frequency image sequence by different gain coefficients. This aspect has already been explored in [21]. Since the pixels whose high-frequency coefficients are close to zero in the underlying high-frequency image basically correspond to some small noises in the original image. If the gain curve in the above literature is used to enhance the image, these fine noises in the image will be seriously amplified. The enhancement curve of the following form is given in [21]:

(12) $$y(x)=\left\{ \begin{array}{ll} aM\frac{x}{x_{c}}{\left(\frac{x_{c}}{M}\right)}^{p} & \;if\;\;\;\;\;\;\;\;\left|x\right|<x_{c}\\ aM\frac{x}{\left|x\right|}{\left(\frac{\left|x\right|}{M}\right)}^{p} & \;if\;\;\;\;\;\;\;\;\left|x\right|>x_{c} \end{array}\right.$$

where $-M<x<M,\;0<x_{c}<<M$; p is a parameter that controls the degree of nonlinearity of the curve and also controls the maximum gain of the image; M is the maximum gray absolute value of the high-frequency image; and a controls the minimum gain of the image.

**Figure 5:**
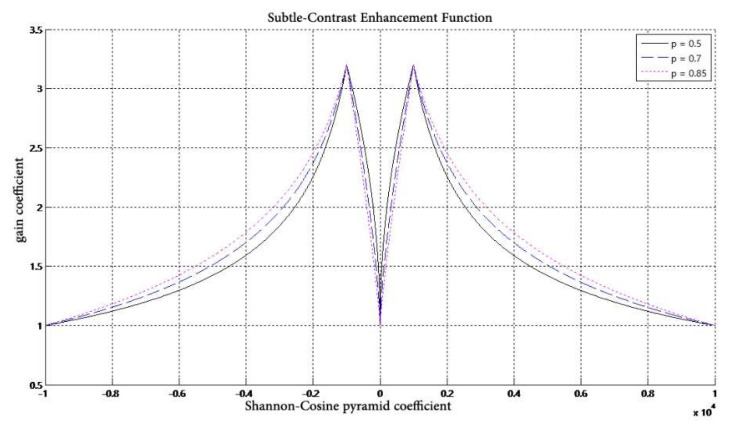
Improved gain function.

We therefore improve the gain curve proposed in [21] in order to increase the contrast of the finest details in the image while suppressing the fine noise in the image, as shown in Figure 5. Such a curve is necessarily a curve that rises sharply to a maximum at the far point and then falls slowly. We suppress the part of the gain curve in [21] where the absolute value of the coordinate is less than $X_{C}$. We thus enhance without overamplifying the noise.

In Figure 5, the degree of freedom, p, is the parameter that controls the degree of the contraction of the nonlinear gain curve and also controls the maximum gain value of the image. However, such a curve is a change of a jumping nature due to the discontinuity at the peak. It can lead to an image in which the gain at the peak is too strong and the gain near the peak is not strong enough, and it can even happen that the region that was originally of strong contrast becomes a region of weak contrast. For the above considerations, the following form of gain curve is proposed:

(13) $$y=\left|x/(A+Bx^{2})\right|$$

**Figure 6:**
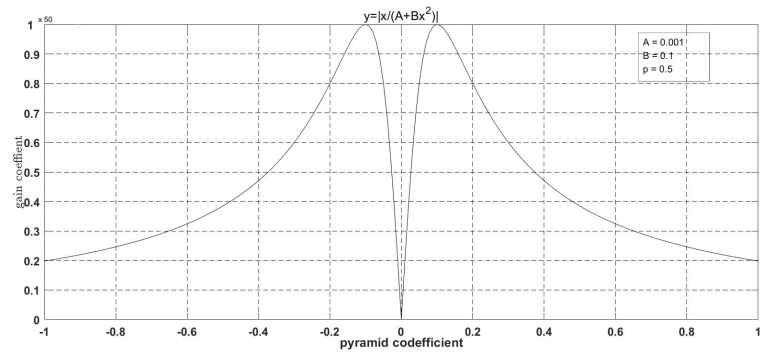
Basic form of the proposed gain function.

The image corresponding to this curve is shown in Figure 6.

The horizontal coordinates of the curves in Figure 6 represent the coefficients of the high-frequency images in the normalized detail space (i.e., the pixel gray values of the high-frequency images), and the vertical coordinates represent the gain values corresponding to the various high-frequency coefficients. The advantage of the curve is that it can control the peak, $y_{max}$, of the curve and the horizontal coordinate corresponding to the maximum, $y_{max}$. However, because it has only two degrees of freedom, this curve does not well control the degree of nonlinearity of the curve rise and decay. Therefore, we add another variable, *C*, to the metric, such that the functional form becomes:

(14) $$y=x/(A+Bx+Cx^{2})$$

This function has three degrees of freedom, and by comparing it with (13), it is no longer an even function, so now we can control a different decay for an increasing value of *x*. By finding the extreme value of the function, it is easy to derive coordinate $x_{\max}=\sqrt{A/C}$ when the maximum value is taken. The maximum value is $y_{\max}=1/(2\sqrt{A\cdot C}+B)$ and the boundary value is $y_{x=1}=\frac{1}{A+B+C}$. From this, the restrictive relationship between the three variables of *A*, *B* and *C* can be obtained as follows.

(15) $$C=\frac{1/y_{\max}-B}{2\cdot x_{\max}}$$

(16) $$A=x_{\max}^{2}\cdot C$$

where *B* can be freely selected to adjust the degree of curve attenuation. In this way, when *B* is selected, both the maximum value of the curve and the horizontal coordinate corresponding to the curve at the maximum value can be kept constant while the degree of nonlinear decay of the curve changes. By using basic mathematical analysis, it can be concluded that the smaller the value of *B* curve decay is faster. The curve finally decays to:

(17) $$y_{x=1}=\frac{1}{A+B+C}=\frac{2\cdot x_{\min}}{(x_{\min}^{2}+1)/y_{\min}-{\left(x_{\min}-1\right)}^{2}\cdot B}$$

We can also use another form of curve with three degrees of freedom:

(18) $$y=\frac{x}{A+B\cdot x^{1+p}}$$

The constraints on A, B, and p are calculated from the corresponding coordinates, $x_{\max}$, and from the extreme values at the peaks, as follows:

(19) $$A=p\cdot B\cdot x_{\max}^{p+1}$$

(20) $$B=\frac{1}{(p+peak)x_{\max}^{p}}$$

**Figure 7:**
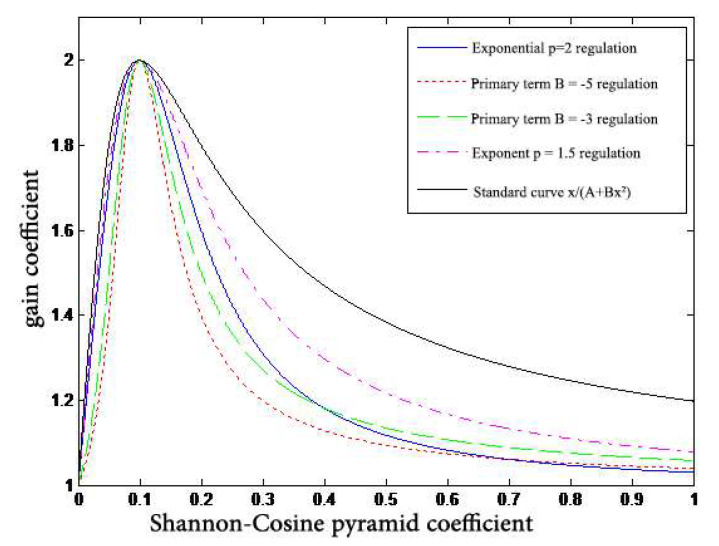
Comparison of two model curves.

Figure 7 shows a comparison of the attenuation effect of the two models.

The disadvantage of these two curves is that the linearity of the decay is not well controlled. The reason is that the degree of the nonlinear decay of the curve and the final point of the curve make up a pair of irreconcilable contradictions. The solution is to add another degree of freedom to the formula, which can control the degree of the nonlinearity of the decay while fixing the decay point of the curve.

To achieve this, we combine the two models and add another degree of freedom, p, to the function. The form of the curve then becomes:

(21) $$y=\frac{x}{A+B\cdot x+C\cdot x^{p}},\;x>0$$

**Figure 8:**
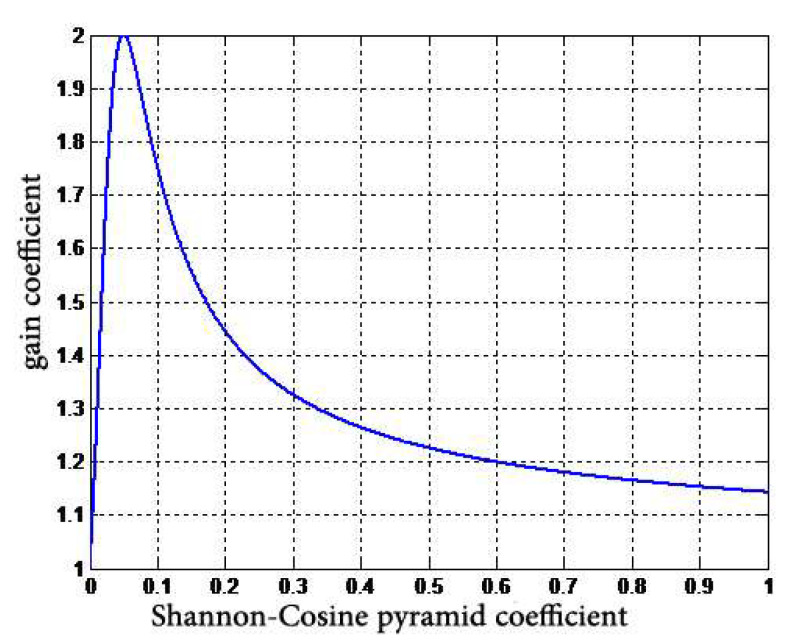
Proposed gain curve.

The curve is shown in Figure 8.

The constraint relationship between A, B, C, and p is solved according to the fixed peak and the corresponding coordinate, $x_{\max}$, at the peak, as follows:

(22) $$A=f(C,p)=C\cdot (p-1)\cdot x_{\max}^{p}$$

(23) $$C=g(B,p)=\frac{1/peak-B}{p\cdot x_{\max}^{p-1}}$$

In Figure 8, in coefficients p = 1.5 and B = −3, B and p are used as two input coefficients to adjust the drop point of curve decay and the decay amplitude.

In practice, different curves and different coefficients are used for each layer of high-frequency images on the basis of empirically based noise estimation. The curves in [21] or [22] are used directly at the coarser scales because of the continuous low-pass filtering in the pyramid decomposition algorithm, which is already almost free of noise interference at the coarser scales.

Of course, there are many other functions available, such as $y={\left[x^{\beta }/(x^{\beta +\alpha }+\sigma^{\beta })\right]}^{1/j}$, and certain segmented curves [23], which are mostly not very controllable. In addition, because of the discontinuity of the first-order derivatives of certain gain functions, the enhancement generates some spurious information.

### 3.2. Adaptive Multiscale Noise Reduction in DR Images

The multiscale enhanced denoising algorithm based on Shannon–Cosine wavelet divides the image into several sub-images, from high frequency to low frequency, so that different scales naturally correspond to different details and structures at different sizes in the original image. The highest-frequency sub-image corresponds to the finest detail of the original image, the lowest-frequency sub-image corresponds to the rough description of the original image, and so on. Then different detail images are processed separately, to achieve the simultaneous enhancement of different sizes among the details in the image.

The main steps of our method are as follows. First, the decomposed level-3 detail image is used to calculate the neighborhood standard deviation of each pixel in the image to generate an image recording the activity level of each pixel. The reason for choosing level 3 is that, in general, the image decomposition is almost noiseless after level 3 because of continuous Gaussian smoothing during the decomposition process. Nevertheless, we performed low-pass filtering on the level-3 image to further reduce the noise effect. The smoothed image was then used to calculate the activity level, $v_{i}$, of each pixel on the basis of the activity level in the image measured as the local standard deviation over a 3 × 3 pixel block given by:

(24) $$v_{i}(n,m)=sqrt\left(\frac{1}{9}\sum_{i=n-1}^{n+1}\sum_{j=m-1}^{m+1}{\left(x(i,j)-\bar{x}(n,m)\right)}^{2}\right)$$

where $\bar{x}(n,m)$ is the average luminance level over the same 3 × 3 pixel support.

After the activity of each pixel is calculated, the pixel activity is interpolated to correspond to each level of the Shannon–Cosine wavelet sequence. In order to better eliminate boundary artifacts, mainly the bilinear interpolation algorithm is used. The noise attenuation is performed in each level of the image according to the strength of each pixel’s activity. Pixels with particularly low activity basically correspond to the smooth part of the image and can be attenuated for the purpose of noise removal, those with medium activity correspond to the less distinct structures in the image and are maintained, and those with maximum activity correspond to the more distinct structures and are maintained or enhanced. In practice, we attenuate the noise only in the first three levels of the Shannon–Cosine wavelet sequence, 0, 1, and 2, because the noise is largely eliminated in the subsequent levels because of multiple instances of Gaussian smoothing.

## 4. Multiscale Medical-Image-Enhancement and Noise-Reduction Simulation

### 4.1. Simulations for Multicale Diagnostic Detail Enhancement on DR Images

**Figure 9:**
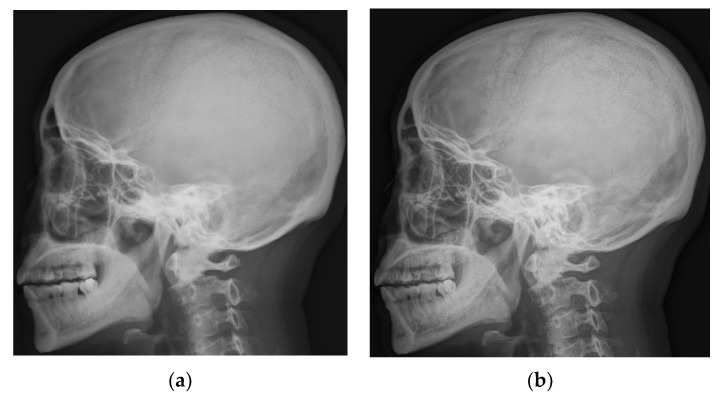
Comparison between the proposed method and the method in [22]. (**a**) The results obtained using the gain curves in [22]. (**b**) Results obtained using the gain curves proposed in this paper.

The gray values of different pixels in the high-frequency image sequence are multiplied with the gain values obtained by the two methods, and the results are shown in Figure 9, which shows the effect of the gain curve obtained by Equation (12) and the curve generated by the improved algorithm of this study, namely Equation (21), on the tone-adjusted image. Our curve has a more-obvious enhancement effect on the details. The curve before the improvement is too large for noise amplification, resulting in subtle details’ being covered up, especially some subtle textures in the skull and structures in the cranial cavity, which will affect a doctor’s diagnosis.

**Figure 10:**
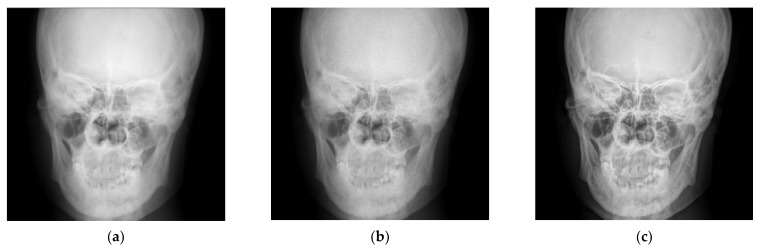
Comparison between the proposed method and the classical Sigle-scale unsharp masking method. (**a**) The head image after hue transformation. (**b**) Processing result of classical single-scale inverse sharpening mask algorithm. (**c**) The enhancement algorithm proposed in this paper.

Figure 10 shows a comparison between the algorithm proposed in this paper and the classical single-scale inverse sharpening mask algorithm.

Figure 10a shows a cephalometric image, which was processed using the classical single-scale inverse sharpening mask algorithm and the algorithm proposed in this paper, separately. There are various measures of image quality, and because everyone has a different understanding of image quality, the subjective visual effect is compared here mainly from the physician’s diagnostic point of view. Figure 10b shows the processing results of the classical single-scale inverse sharpening mask algorithm, and Figure 10c shows the processing results of the enhancement algorithm proposed in this paper. Figure 10 shows that the image after processing by the inverse sharpening mask algorithm has clearer texture details than the original image, but at the same time, the noise is larger, especially that of the top of the head. Although the inverse sharpening mask algorithm provides a certain improvement on the detail, at the same time it more seriously amplifies the noise. The processing result of the algorithm proposed in this paper is clearer in both the texture of the top of the skull and the details near the nasal cavity, and the noise is much smaller than that of the inverse sharpening mask algorithm.

### 4.2. Simulations for Multiscale Noise Reduction in DR Images

The visual characteristics of the human eye determine that the human eye is much more sensitive to noise in smooth regions than in structured regions. Any form of noise reduction will have some impact on the image quality and even bring some false information. Especially in the case of diagnostic-related structures, improper noise reduction may bring the danger of a misdiagnosis. Therefore, noise attenuation should be conducted with extreme caution.

**Figure 11:**
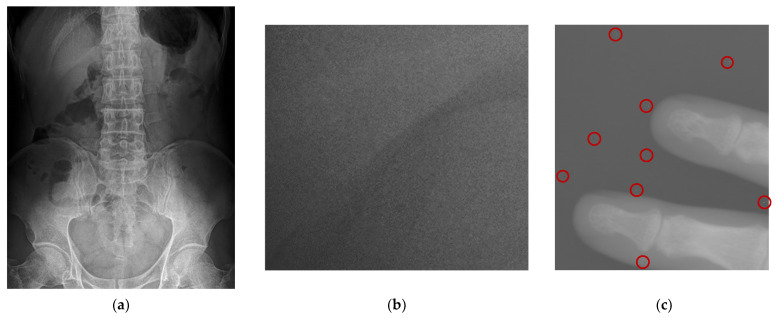
Noise in digital radiography image. (**a**) Enhanced abdominal image with uniform fine particles (**b**) Enlarged detail section (**c**) Raw DR image of a local hand with speckle noise.

While noise may exist in different frequencies of information, the most important noise for diagnosis exists in the high-frequency domain. Because of the characteristics of DR imaging, the noise points are generally particularly uniform fine particles of impulse noise and some particularly obvious speckle noises, as shown in Figure 11.

For the noise in Figure 11c, it is more remarkable that there is a clear, abrupt change in the local neighborhood. In addition, only a few points in the local neighborhood of the speckle do not differ much from its gray scale. According to this characteristic of this speckle noise, we have designed the following algorithm to remove such noise:

1. Set the amplitude, a, of the speckle pulse and the size, S, of the local neighborhood.

2. Perform a point-by-point scan of all pixels in the image. Mark the currently scanned pixel as the center pixel, c.

3. Count the number, n, of neighborhood points whose absolute value of difference from the central pixel, c, is greater than a, and record the set, E, of those neighborhood points whose absolute value of difference from the central pixel is greater than a.

4. If n is greater than a predetermined threshold, thr, then the gray-scale value of that central pixel, c, is set to the average or statistical median of the pixels in the set (S-E).

Figure 10 shows the original localized digital X-ray image of the hand and the result after removing speckle noise by using the algorithm in this paper.

**Figure 12:**
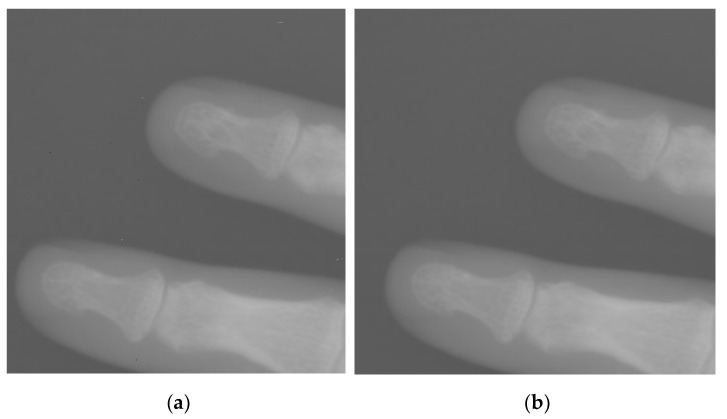
Speckle noise removal from localized hand X-ray radiographic images. (**a**) Before removal of speckle noise (**b**) After removal of speckle noise.

**Figure 13:**
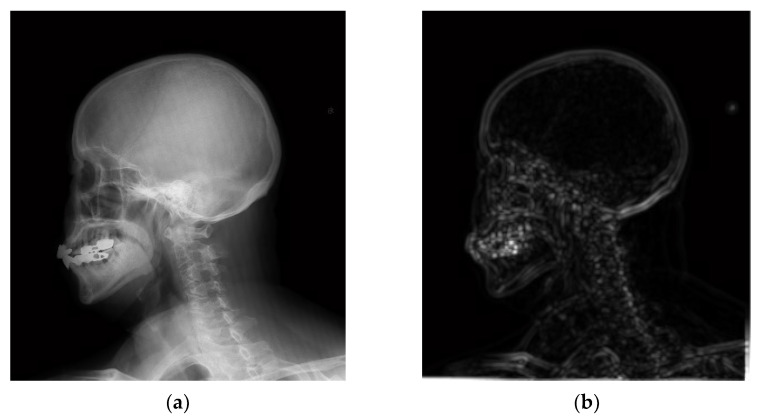
Comparison between the original image and an image of pixel activity. (**a**) Original image (**b**) Pixel-activity map calculated by the meth-od in this paper.

Figure 12 illustrates the speckle noise rejection algorithm proposed in this paper. As long as the appropriate initial coefficients have been set, those isolated speckle noises can be well suppressed, without losing the details in the image. Because the local fluctuations in the smoothed region are not large, the response to the mathematical concept is that the variance in the local neighborhood is small. In addition, the local fluctuation of the detail region is larger, which is reflected in the mathematical concept that the variance within the local neighborhood is larger. We define the local intra-neighborhood variance or standard deviation of a central pixel as the active degree of that pixel, and the active degree of a pixel can also be replaced by a mathematical concept such as entropy for the local neighborhood variance. According to the activity level of each pixel in the DR image, the activity level of the pixel is bilinearly interpolated, and the noise is attenuated according to the intensity of the activity level of the pixel. Figure 13 shows a comparison between a skull image and a pixel-activity-level image.

**Figure 14:**
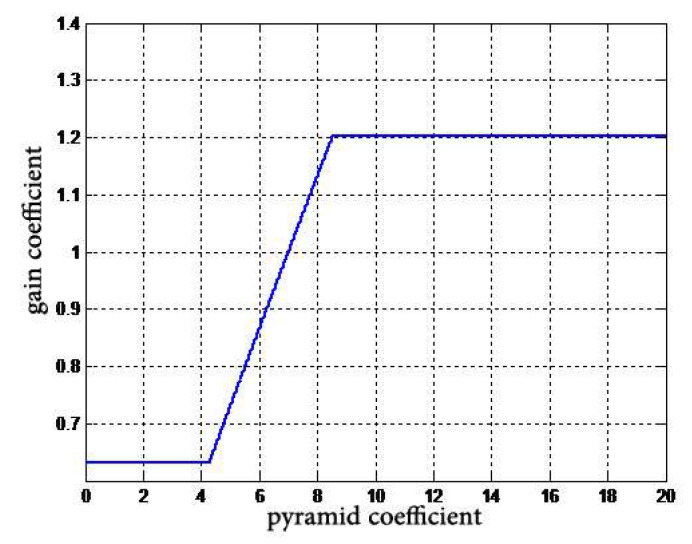
Schematic of noise attenuation curve.

The attenuation curve is schematically shown in Figure 14. The key points of attenuation and gain are set according to the noise level of the DR image acquisition device. Then three-line segments are used to splice the curve of noise attenuation.

**Figure 15:**
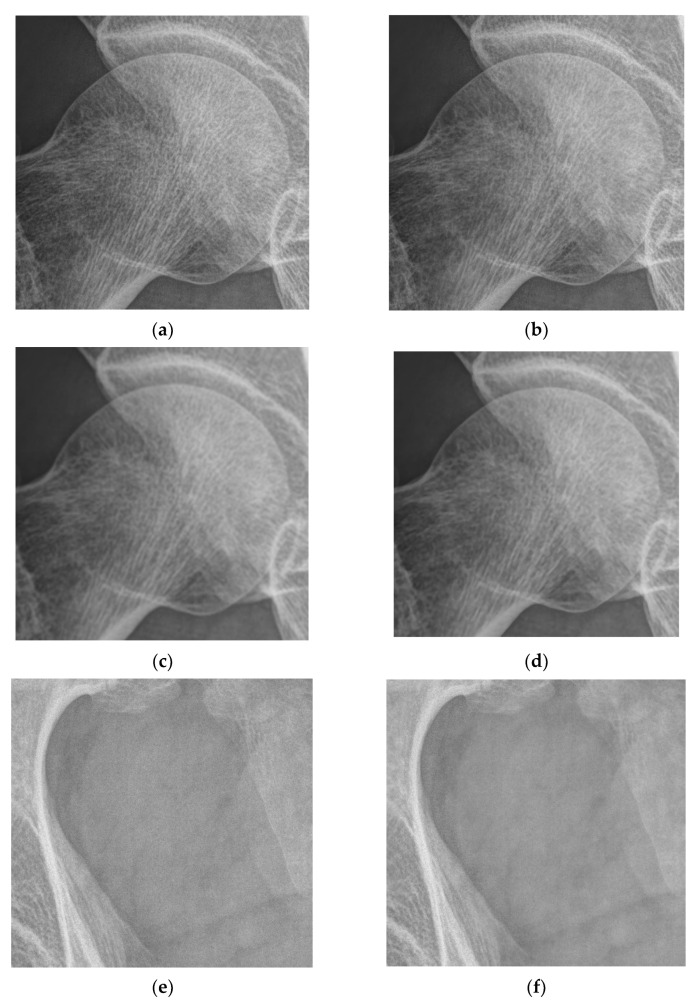

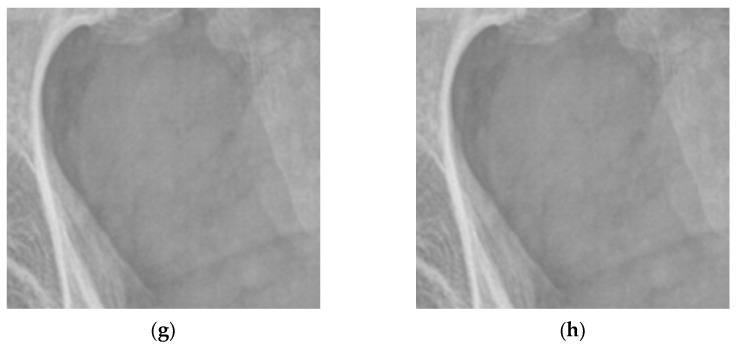
Comparison between the proposed denoising method, neighbor averaging filtering, and median filtering. (**a**) The magnified bone texture part (**b**) After noise reduction by the algorithm in this paper (**c**) After mean filtering process (**d**) After median filtering process (**e**) The smoothed area of the magnification (**f**) After processing by the algorithm in this paper (**g**) After mean filtering (**h**) After median filtering.

Figure 15 shows the comparison of the proposed algorithm in this work with the classical median filter and the classical average filter.

As can be seen in Figure 15, the classical filtering method loses details in the bone texture and in important structures when filtering the smoothed region. In addition, the method proposed in this paper can keep the detail region in the DR image and suppress the noise content of the smoothing threshold.

In the work, artificial noise (pretzel noise with an intensity of 0.001 and Gaussian noise with mean and variance of 0 and 0.001, respectively) was added to the original image to conduct a quantitative comparison. The peak signal-to-noise ratio (PSNR) and structural similarity (SSIM) index of the noise-reduced enhanced images obtained by different methods are given in Table 1. These two parameters are commonly used indicators to evaluate image quality. The enhancement and denoising effects of different methods when the noise content is increased are given in Table 1.

An analysis of the results presented in Table 1 indicates that among various noise-reduction methods, when the two parameters of PSNR and SSIM are compared, although the SSIM index CLAHE algorithm achieved the best calculated result of 0.8426, which is 0.0129 higher than the algorithm of this study, the result of PSNR shows that the algorithm of this paper has a clear advantage and obviously achieved a good result of 36.9548.

## 5. Conclusions

Current research has provided a viable and stable algorithm for digital X-ray image enhancement, which has been implemented by using specific development tools. However, a mature medical-image-processing algorithm often requires a long refinement process and needs to be based on a large number of clinical experiments. The tone-curve-generation algorithm discussed in this paper results only in an improvement of the overall contrast of the image without adjusting for the most likely areas of disease, and in a few cases, the tone adjustment of some images is less than optimal. Adaptive tone-curve adjustment can take advantage of the diagnostic information that a doctor needs on different tissues in different parts of the body to better highlight the parts that are relevant to the diagnosis. On the other hand, the multiscale noise-reduction part of the DR image can also incorporate some kind of noise-estimation mechanism to more appropriately select the threshold for noise attenuation and conduct adaptive image noise reduction. In addition, the tone-curve-generation algorithm studied in this paper is only for linear A/D conversions, and more-suitable algorithms can be investigated for images using different types of A/D conversions.

DR image post-processing plays a significant role in the diagnosis of medical personnel. In this paper, a systematic and in-depth study of existing DR image post-processing algorithms was conducted, and a multiscale image noise-reduction and multiscale image-enhancement algorithm based on Shannon–Cosine wavelet transform decomposition was proposed according to the characteristics of DR images. Through visual comparison, it was shown that the algorithm can achieve better results than the traditional inverse sharpening mask algorithm in terms of both detail enhancement and noise suppression.

## Figures and Tables

**Table 1:** Objective indicator of image-enhancement and noise-reduction quality.

Algorithms	PSNR	SSIM
HE	28.8677	0.6946
CLAHE	32.2087	0.8426
Wavelets	30.4563	0.5261
This study	36.9548	0.8297
